# Emerging and reemerging respiratory viral infections up to Covid-19

**DOI:** 10.3906/sag-2004-126

**Published:** 2020-04-21

**Authors:** İlhami ÇELİK, Esma SAATÇİ, Füsun Öner EYÜBOĞLU

**Affiliations:** 1 Department of Infectious Diseases and Clinical Microbiology, Kayseri Health Practice and Research Center,University of Health Sciences, Kayseri Turkey; 2 Department of Clinical Microbiology, Kayseri Health Practice and Research Center, University of Health Sciences, Kayseri Turkey; 3 Department of Pulmonary Diseases, Başkent University, Ankara Turkey

**Keywords:** Emerging, reemerging, respiratory virus, Covid-19

## Abstract

Infectious diseases remain as the significant causes of human and animal morbidity and mortality, leading to extensive outbreaks and epidemics. Acute respiratory viral diseases claim over 4 million deaths and cause millions of hospitalizations in developing countries every year. Emerging viruses, especially the RNA viruses, are more pathogenic since most people have no herd immunity. The RNA viruses can adapt to the rapidly changing global and local environment due to the high error rate of their polymerases that replicate their genomes. Currently, coronavirus disease 2019 (COVID-19) is determined as an u28a9 caused by severe acute respiratory syndrome coronavirus 2 (SARS-CoV-2), which was first identified in 2019 in u28ac. Herein we discuss emerging and reemerging respiratory viral infections till to SARS-CoV-2.

## 1. Introduction

Infectious diseases that increase in incidence and tend to spread geographically within decades can be defined as emerging infections. Pathogens of these infections appear for the first time or they have existed previously and spread rapidly among the population and new geographical areas. The emergence of novel human pathogens and reemergence of several diseases is of particular concerns of the current century [1]. There is a dominance of zoonotic infections, mostly originating in wildlife, among emerging health threats with a rate of 70%. Pathogens first emerge in themselves and rapidly mutate, which result in a transmission in humans with subsequent dissemination. According to the extent of the transmission, epidemic outbreaks may occur and progress to a pandemic. Diseases that reappear after a significant decline are called as reemerging diseases. Reemergence may occur due to a breakdown in public health measures or the appearance of new strains of organisms [2]. Respiratory infections with epidemic and pandemic potential that cause a global burden have plagued people since the beginning of human history. In this review, the epidemic and pandemic, emerging and reemerging respiratory viruses are summarized in Table. 

Most emerging viruses come from animals and are zoonotic or vector-borne diseases belonging to the families *Orthomyxoviridae*, *Paramyxoviridae*, *Picornaviridae*, *Coronaviridae*, *Adenoviridae*, and *Herpesviridae*. Community-acquired respiratory viruses are critical pathogens such as influenza, respiratory syncytial virus, adenovirus, parainfluenza virus, human coronavirus, human metapneumovirus, rhinovirus, enterovirus, cause millions of deaths and hospitalizations around all over the world every year [3,4]. 

In the last century, influenza originated avian, and swine, severe acute respiratory syndrome-coronavirus (SARS-CoV) and the Middle East respiratory syndrome-coronavirus (MERS-CoV) were the most damaging respiratory infections for human being all over the world [5]. These emerging viral respiratory infections derived from the animal world [6]. Mutations in the genetic material of RNA viruses accumulate in years and produce new strains of the viruses with new antigenic properties resulting in a transmission in humans [7]. The probability of pandemics with new viruses would be high in the future as this type of mutations will reoccur. 

The other mechanism of a virus is reassortment which means that the host is infected with 2 different strains of viruses (animal and human viruses) than a new generation of a new virus with mixed genetic materials is developed and causes new pandemics [7]. Bird and swine influenza viruses obtain new gene segments through a reassortment with human strains.

**Table 1:** Outbreaks of emerging and reemerging respiratory viral infections.

Virus	Year	Region
Spanish Flu H1N1	1918	Spain [25]
Asian flu H2N2	1956	East Asia [28]
HCoV-229E HCoV-OC43	1960	The different part of the World [29]
Hong Kong Flu H3N2	1968	Hong Kong [28]
Hantavirus pulmonary syndrome	1993	USA [16]
Influenza A H5N1	1997	Hong Kong [28]
Influenza A H9N2	1999	Hong Kong [28]
Human metapneumovirus	2001	Netherlands [18]
SARS CoV	2002–2003	Guangdong, China [22]
Human CoV NL63	2004	Netherlands [20]
Influenza A H7N7	2004	Netherlands [28]
Human CoV HKU1	2005	China [20]
Triple reassortant H3N2 Influenza A	2005	Canada [28]
Bocavirus	2005	Sweden [19]
Influenza A H1N1 pmd09	2009	Mexico [28]
Adenovirus 14	2010	USA [30]
Influenza (H3N2)v	2011	USA [9]
MERS-CoV	2012	Saudi Arabia [23]
Influenza A H7N9	2013	China [28]
Influenza A H10N7	2014	China [28]
SARS-CoV-2	2019	China [26]

The mutation in the genetic material of RNA-virus also sometimes drifts in time and builds a chimeric virus, containing nucleic acid fragments or proteins from 2 or more different viruses. This novel hybrid virus is different from parental viruses [8]. 

Currently, COVID-19 caused by a zoonotic virus u2959 (SARS-CoV-2) was first determined in 2019 in u295c, the capital of China’s u295d province. Epidemiologic data show exposures of some initial cases in Huanan seafood and live animal market and early phylogenetic results suggest initial human infection in November 2019 followed by ongoing human-to-human transmission. Animal source has not been identified yet; and COVID-19 has since spread globally, resulting in the current u295e. The majority of cases (approximately 96%) occur with mild respiratory symptoms, some progress to u295f, acute respiratory distress syndrome, respiratory insufficiency and u2960. The overall u2961 is 4.6%; ranging from 0.2% to 15% according to age group and u2962. 

## 2. Emerging and reemerging viruses in the last century

### 2.1. Influenza viruses

The influenza viruses are the world’s most critical epidemic viruses. Influenza pandemics occurred earlier in 1918 (swine flu), 1957 (Asian flu), 1968 (Hong Kong flu), 1977 (Russian flu), and the most recent pandemic in 2009 (pandemic influenza A H1N1) [9]. It belongs to the *Orthomyxoviridae* family. Influenza is an enveloped virus with negative-stranded RNA consists of 8 segments. There are 4 different types; A, B, C, and D [7, 10]. Influenza A virions possess 2 surface glycoproteins—the hemagglutinin (HA) and neuraminidase (NA)-which exert different functions. Eighteen hemagglutinin and 11 neuraminidase subtypes are known to exist in nature [10]. They can infect birds and mammals, including man. Influenza B is restricted to human horde. Influenza C is isolated from humans, pigs and dogs [10]. Influenza D viruses primarily affect cattle and human population in all age groups [11]. Seasonal influenza viruses kill 250,000–500,000, mostly older people each year around the world [3]. 

### 2.2. H1N1 influenza

Influenza virus type A is very variable and shows a continuous antigen variation. It is a significant cause of epidemics and pandemics. The surface antigenic glycoproteins are subject to 2 main types of antigenic variation, namely: antigenic shift and antigenic drift. The antigen shift is an abrupt, significant change in an influenza A virus that leads to new HA and/or new HA and NA proteins in influenza viruses that infect man. The interruption can lead to a novel influenza A subtype in humans. These are small changes (or mutations) in the genes of influenza viruses that can guide to alterations in the surface proteins of the virus: HA (hemagglutinin) and NA (neuraminidase). The HA and NA surface proteins of influenza viruses are “antigens”, which implies that they are seen by the immune system and can activate an immune response, including the production of antibodies that can halt the contagion. The changes associated with antigen drift occur continuously over time as the virus replicates. Most flu shots target the HA surface proteins/antigens of an influenza virus.

The most fatal and unforgettable outbreak “mother of the pandemic” virus occurred in 1918 named as Spanish influenza. In this eruption, approximately 50 million people [12, 13] were dead. H1N1 was occurred in different years (1928, 1932, 1936, 1943, 1947) during this century [7].

In 1977, H1N1 was reemerged and named as Russian flu, which mostly affected young people. In 2009, H1N1 was the reason of a new pandemic. It was first detected in the USA. This one delivered an unparalleled combination of influenza genes with a triple reassortment [7].

### 2.3. H2N2 influenza 

In 1957, a new strain appeared in the world named H2N2, emerged in humans in Southeast Asia and rapidly spread worldwide. The virus persists in wild and domestic birds. The reemergence of H2N2 in humans is a significant threat due to the absence of humoral immunity, and it was the case of the second pandemic of the 20th century [14]. 

### 2.4. Avian influenza (AI)

Humans are susceptible to avian influenza virus subtypes-A (H5N1), A (H7N9) and A (H9N2). Exposure to infected birds or contaminated environment is thought to underlie human infection with these viruses. There have been sporadic cases of human infections with AI and other zoonotic influenza viruses, but sustained human-to-human infection and transmission have been lacking. Although the public health risk from the currently known influenza viruses at the human-animal interface remains the same, the sustained human-to-human transmission of this virus is low. Avian influenza A viruses (AIVs) are among a terrifying emerging and reemerging pathogen because of their possible risk of causing an influenza pandemic. The growth in domestic animals and poultry worldwide is followed by the ascent of human AIV outbreaks [3].

The risk of death is highest among reported cases infected with H5N1, H5N6, H7N9, and H10N8 infections. Senior people and males tended to take in a lower hazard of infection with most AIV subtypes, except for H7N9. Visiting live poultry markets were generally reported by H7N9, H5N6, and H10N8 cases, while exposure to sick or dead bird mostly reported by H5N1, H7N2, H7N3, H7N4, H7N7, and H10N7 cases [15].

### 2.5. Hantavirus 

Hantavirus pulmonary syndrome (HPS) has emerged from the infection with *Sin Nombre virus *(SNV). It is a negative-sensed, single-stranded RNA virus. The virus was first identified in the United States in 1995. Since then, 71 people were diagnosed as HPS, and all patients were California residents.* *Persons were usually exposed to SNV through inhalation of aerosolized excreta (e.g., saliva, urine, and faeces) from infected wild animals (rodents, typically deer mice). HPS has demonstrated a severe disease characterized by pulmonary oedema, followed by respiratory failure than cardiogenic shock [16,17]. 

### 2.6. Human metapneumovirus (HMPV) 

HMPV is a negative-sense, single-stranded RNA virus of the family Pneumoviridae, cause of acute respiratory infection, particularly in children, immunocompromised patients, and the aged. HPMV genome was mostly similar to avian metapneumovirus serotype C. In 2001, researchers in the Netherlands identified HMPV from nasopharyngeal samples from 28 children. The virus is a leading cause of acute respiratory infection, particularly in children, immunocompromised patients, and the elderly. Almost every child is infected with HMPV by the age of 5 [18]. 

### 2.7. Bocavirus

Human bocavirus (HBoV) is a member of the*Parvovirus* (DNA) family, detected in 2005. It is distributed worldwide, and evidence indicates that HBoV is the cause of the respiratory tract infections. Numerous studies demonstrate HBoV as a co-pathogen. Studies prove that its prevalence in asymptomatic patients is high [19].

### 2.8. Coronavirus

The *Coronaviridae* family includes a broad spectrum of animal and human viruses, with typical morphology. Before 2003, the virus family was known as the cause of only mild respiratory illness in humans. However, the emergence of severe acute respiratory virus (SARS-CoV) and MERS-CoV shows the zoonotic potential of causing severe disease outbreaks in humans [20]. 

Coronaviruses also have the largest positive-sense RNA genome, which is expressed by a complicated procedure. This genomic type allows the formation of encrypting RNA transcripts in a genome. Somehow, encrypted sequences are progressing during the replication cycle and produce new types of coronavirus. Coronaviruses are classified into 3 groups, grounded on the antigenic properties of virus proteins: the spike (S), membrane (M), and nucleocapsid (N) proteins [20].

### 2.8.1. HCoV-229E and HcoV-OC43

Human virus HCoV-229E is one of the species in genus alphacoronavirus, and it is the first group. It is the first coronavirus, isolated from patients with upper respiratory tract infections in the 1965. Until late 2002, only 2 human coronaviruses were very well known–HCoV-229E and HCoV-OC43. HCoV-OC43 is from the genus betacoronavirus, and it is in the second group of coronaviruses. HCoV-OC43 also isolates from patients with upper respiratory tract infections.

HCoV 229E and OC43 are the known causes of the common cold within the last 200 years. They are now globally endemic in humans, crossed species from their animal reservoirs (bats and cattle, respectively) to humans [20]. 

### 2.8.2. Severe acute respiratory syndrome coronavirus (SARS-CoV) 

Severe acute respiratory syndrome (SARS) has emerged initially from ancestral bat viruses. SARS-CoV was the ﬁrst known major pandemic coronavirus. The disease occurred in late 2002 when an outbreak of acute community-acquired atypical pneumonia syndrome was first diagnosed in Guangdong Province, China. Over 8000 people were affected, with a crude fatality rate of 10% in more than 12 countries [21].

The WHO issued a worldwide warning about the disease on March 13, 2003. Although the cases generally remained confined to China, a few cases were reported from North and South America, Europe and Asia. Luckily, new cases with SARS has not been described since 2004 [22].

### 2.8.3. Middle east respiratory syndrome coronavirus (MERS-CoV)

MERS-CoV is a zoonotic viral disease that causes respiratory infection. It was first reported in Saudi Arabia in 2012 and has expanded to 26 different countries. Since 2012, over 2207 laboratory-confirmed cases and 787 deaths from MERS-CoV infection have occurred worldwide.

The clinical spectrum of illness associated with MERS-CoV ranges from asymptomatic infections to acute respiratory distress syndrome, resulting in multi-organ failure and mortality. The case-fatality rates have stayed high at 3–4 per 10 cases [23]. 

Francis and his colleagues (2007) were mentioned around the emergency of newly reemerging coronaviruses. Due to coronaviruses genetic recombination, newly emerged ones may go to new genotypes and outbreaks. They specially indicated the reservoir (horseshoe bats) of SARS-CoV-like viruses as a source of novel types of coronaviruses, because of the culture of eating exotic mammals in southern China. They said that it is a time bomb of the possibility of the reemergence of SARS [20]. 

### 2.8.4. SARS-CoV-2

In December 2019, a novel coronavirus (SARS-CoV-2) was observed in 3 patients with pneumonia connected to the cluster of acute respiratory illness cases from Wuhan, China. By the end of March 2020, the virus spread all over the globe and caused a large global outbreak [24]. On January 30 2020, the WHO declared the COVID-19 outbreak as the sixth public health emergency of international concern, following H1N1 (2009), polio (2014), Ebola in West Africa (2014), Zika (2016) and Ebola in the Democratic Republic of Congo (2019) [25,26]. WHO announced a COVID-19 outbreak as pandemic on Mar 12, 2020. 

SARS- CoV-2 was found to be a positive-sense, single-stranded RNA virus belonging to the genus Betacoronavirus, closely related to 2 bats-derived severe acute respiratory syndrome-like coronaviruses, bat-SL-CoVZC45 and bat-SL-CoVZXC21 [24,27]. 

Although early studies reported a possible link between animal-to-human transmission, later studies demonstrated human-to-human transmission of SARS-CoV-2 through droplets or direct contact. Besides symptomatic patient transmission, there are shreds of evidence of the possibility of transmission by asymptomatic carriers [26]. 

## 3. Conclusion

The emerging and reemerging respiratory virus infections are a continuing threat to human life [10]. Reassortment, chimerism, antigenic shift, antigenic drift, and other molecular mechanisms cause new epidemic and pandemic of coronaviruses. RNA viruses are much more vulnerable to a chromosome mutation mechanism [7,10] as an outcome of ecological change [10]. Human-animal interface [31], changing environment and tropical deforestation [32], rapidly increasing population, increasing international travel, immigrants and poverty enhance the spread of viral disease around the world [33].

## Acknowledgments

İlhami Çelik and Füsun Öner Eyüboğlu are the members of COVID-19 Advisory Committee of Ministry of Health of Turkey.
